# An Ssd1 Homolog Impacts Trehalose and Chitin Biosynthesis and Contributes to Virulence in Aspergillus fumigatus

**DOI:** 10.1128/mSphere.00244-19

**Published:** 2019-05-08

**Authors:** Arsa Thammahong, Sourabh Dhingra, Katherine M. Bultman, Joshua D. Kerkaert, Robert A. Cramer

**Affiliations:** a Department of Microbiology and Immunology, Geisel School of Medicine at Dartmouth, Hanover, New Hampshire, USA; Carnegie Mellon University

**Keywords:** *Aspergillus fumigatus*, cell wall, chitin, pathogenesis, trehalose, virulence

## Abstract

Regulation of fungal cell wall biosynthesis is critical to maintain cell wall integrity in dynamic fungal infection microenvironments. Genes involved in this response that impact fungal fitness and host immune responses remain to be fully defined. In this study, we observed that a yeast *ssd1* homolog, *ssdA*, in the filamentous fungus Aspergillus fumigatus is involved in trehalose and cell wall homeostasis. An *ssdA* null mutant strain exhibited an increase in trehalose levels and a reduction in fungal colony growth rate. In contrast, overexpression of *ssdA* perturbed trehalose biosynthesis and reduced germination of conidia. The *ssdA* null mutant strain was more resistant to cell wall-perturbing agents, while overexpression of *ssdA* increased sensitivity. Overexpression of *ssdA* significantly increased chitin levels, and both loss and overexpression of *ssdA* altered subcellular localization of the class V chitin synthase CsmA. Strikingly, overexpression of *ssdA* abolished adherence to abiotic surfaces and severely attenuated the virulence of A. fumigatus in a murine model of invasive pulmonary aspergillosis. Despite the severe *in vitro* fitness defects observed upon loss of *ssdA*, neither surface adherence nor murine survival was impacted. In conclusion, A. fumigatus SsdA plays a critical role in cell wall homeostasis impacting A. fumigatus-host interactions.

**IMPORTANCE** The incidence of life-threatening infections caused by the filamentous fungus Aspergillus fumigatus is increasing along with an increase in the number of fungal strains resistant to contemporary antifungal therapies. The fungal cell wall and the associated carbohydrates required for its synthesis and maintenance are attractive drug targets given that many genes encoding proteins involved in cell wall biosynthesis and integrity are absent in humans. Importantly, genes and associated cell wall biosynthesis and homeostasis regulatory pathways remain to be fully defined in A. fumigatus. In this report, we identify SsdA as an important component of trehalose and fungal cell wall biosynthesis in A. fumigatus that consequently impacts the host immune response and fungal virulence in animal models of infection.

## Abstract (precis)

The incidence of life-threatening infections caused by the filamentous fungus Aspergillus fumigatus is increasing along with an increase in the number of fungal strains resistant to contemporary antifungal therapies. The fungal cell wall and the associated carbohydrates required for its synthesis and maintenance are attractive drug targets given that many genes encoding proteins involved in cell wall biosynthesis and integrity are absent in humans. Importantly, genes and associated cell wall biosynthesis and homeostasis regulatory pathways remain to be fully defined in A. fumigatus. In this report, we identify SsdA as an important component of trehalose and fungal cell wall biosynthesis in A. fumigatus that consequently impacts the host immune response and fungal virulence in animal models of infection.

## INTRODUCTION

Aspergillus fumigatus is the most common filamentous fungus that causes a wide variety of human diseases ranging from allergic diseases to acute invasive infections. Invasive aspergillosis (IA) in immune-compromised hosts, e.g., patients with hematological malignancies and organ or stem cell transplant recipients, is associated with high mortality (1). Antifungal drugs used to treat IA, e.g., voriconazole and amphotericin B, are associated with undesired side effects, detrimental drug-drug interactions, long therapeutic regimens, and persistence of poor treatment outcomes (2–5). Compounding the difficulty of treating these infections, the emergence of azole-resistant *Aspergillus* infections is increasing globally (6–28). In order to make therapeutic advances against these increasingly common and potentially drug-resistant infections, new antifungal drugs are needed.

One existing antifungal drug target that has not been fully exploited is the fungal cell wall. Consisting mainly of polysaccharides, including β-1,3-glucans, α-glucans, mannan, chitin, and galactosaminogalactan, among others (29–38), the cell wall is a great antifungal drug target as evidenced by the development of the echinocandins that target β-1,3-glucan biosynthesis. Interestingly, the carbohydrates needed to generate β-1,3-glucan and chitin, i.e., glucose 6-phosphate and UDP-glucose, are also important substrates used to generate trehalose, a disaccharide sugar, which is important for fungal germination of conidia, stress protection, cell wall homeostasis, and virulence (39, 40). The canonical trehalose biosynthesis pathway in A. fumigatus consists of two enzymes, TpsA/B (trehalose-6-phosphate synthase) and OrlA (trehalose-6-phosphate phosphatase), and two regulatory enzyme-like subunits, TslA and TslB (39–41). Trehalose biosynthesis is also found in other organisms, including bacteria, plants, and insects, in addition to fungi but is not found in humans (42).

We and others previously observed that proteins involved in trehalose biosynthesis impact cell wall homeostasis in A. fumigatus (39–41). Disruption of the trehalose-6-phosphate phosphatase OrlA leads to perturbations in cell wall integrity as shown by increased sensitivity to the cell wall-perturbing agents Congo red (CR), calcofluor white (CFW), and nikkomycin Z (40). Loss of OrlA attenuates virulence of A. fumigatus in chronic granulomatous disease (xCGD) and chemotherapeutic invasive pulmonary aspergillosis (IPA) murine models. In addition, a regulatory subunit of the trehalose biosynthesis pathway, TslA, is critical for trehalose production and cell wall homeostasis in part through regulation of a class V chitin synthase enzyme, ChsE/CsmA (41). Loss of TslA increased chitin production and altered the subcellular localization of CsmA (41). Pulldown assays performed with TslA as bait identified a physical interaction between TslA and CsmA as well as a putative Saccharomyces cerevisiae Ssd1 homolog, herein called SsdA.

Ssd1p is a multifunctional RNA-binding protein (43, 44) that is important for chromosome stability at high temperature, vesicular trafficking, stress responses, and cell wall integrity in S. cerevisiae (45–49). Ssd1p genetically interacts with Pkc1p and Sit4p, which are important components of the cell wall integrity signaling pathway (48). It has been shown that S. cerevisiae *ssd1* null mutants isolated from both patients and plants are more virulent than wild-type strains in a DBA/2 murine model (50). Similar to results observed with the A. fumigatus *tslA* null mutant, the cell wall composition of yeast *ssd1* null mutants includes more chitin and mannan with decreases in β-1,3-glucan and β-1,6-glucan levels (50). These cell wall changes lead to increased proinflammatory responses against *ssd1* mutant strains (50). In contrast to S. cerevisiae, the *ssdA*/*ssdA* null mutant in C. albicans has decreased virulence in an invasive systemic candidiasis murine model (51). The function of Ssd1 homologs in filamentous fungal pathogens is less clear, but in the plant pathogen Magnaporthe grisea, *SSD1* is important for fungal colonization of rice leaves (52). Those authors suggested that *SSD1* is essential for proper cell wall assembly leading to evasion of the host immune response (52). However, the mechanism(s) behind this phenotype is not well understood (52). In this study, we characterized a predicted Ssd1p homolog identified in A. fumigatus through its protein-protein interaction with the trehalose biosynthesis protein TslA. Using a genetics approach, we observed that A. fumigatus SsdA is critical for cell wall biosynthesis, trehalose production, polarized growth, biofilm formation, and virulence of A. fumigatus. Our results support the known role of Ssd1 homologs in fungal cell wall biosynthesis and highlight the potential altered functions of A. fumigatus *ssdA*, including those involved in trehalose biosynthesis, biofilm formation, and fungal virulence.

## RESULTS

### SsdA regulates trehalose production and is required for Aspergillus fumigatus germination of conidia and mycelium expansion.

AFUB_010850 was identified in a mass spectrometry-based screen of proteins that interact with the trehalose biosynthesis regulatory protein TslA (41). Protein domain analysis of AFUB_010850 revealed strong amino acid sequence similarity with the nucleic acid binding Interpro domain IPR012340 (5.0E^−109^) and the RNase (RNB) PFAM domain PF00773 (4.4E^−88^). BLASTP analysis of the AFUB_010850 amino acid sequence against the Saccharomyces cerevisiae genome database revealed strong sequence similarity to the protein Ssd1p. Consequently, results of reciprocal BLASTP analyses performed with Ssd1p against the A. fumigatus genome suggested that AFUB_010850 is likely an Ssd1p ortholog and hence we named AFUB_010850 “*ssdA*.” Given the previously identified roles of TslA in trehalose and cell wall homeostasis in A. fumigatus and the known roles of *ssd1* homologs in fungal cell wall biosynthesis, we hypothesized that SsdA is an important mediator of trehalose production and cell wall homeostasis in A. fumigatus.

**FIG 1:**
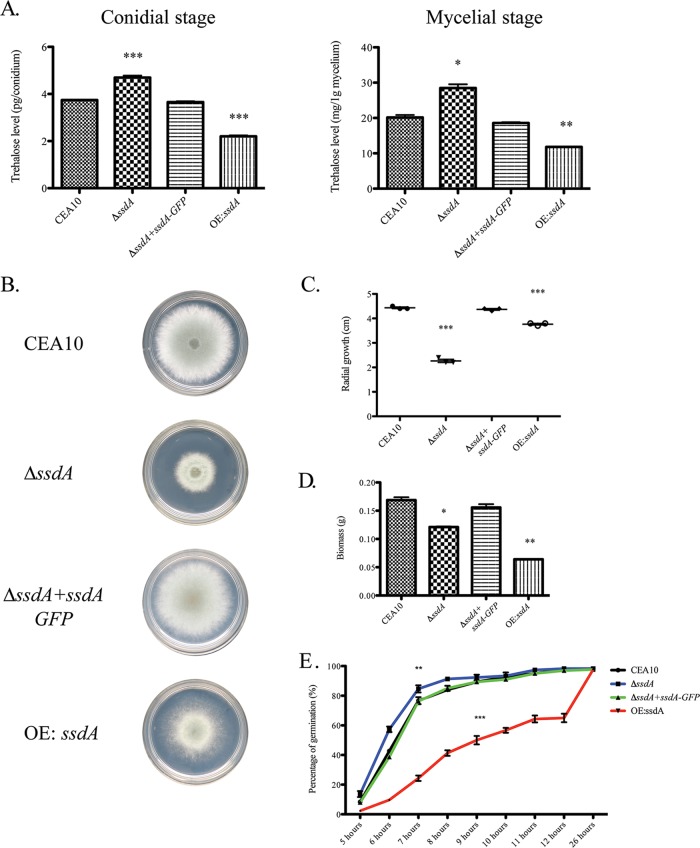
Alteration of *ssdA* expression affects trehalose production, hyphal growth, and germination of conidia. (A) Trehalose assays were performed to measure trehalose levels at both the conidial and mycelial stages using a glucose oxidase assay. Data represent means ± SE of results from three biological replicates. For the conidial stage (left), three asterisks (***) indicate a *P* value of 0.0004 (unpaired two-tailed Student's *t* test) for the comparison of the Δ*ssdA* mutant to the wild type and a *P* value of <0.0001 (unpaired two-tailed Student's *t* test) for the comparison of the OE:*ssdA* mutant to the wild type. For the mycelial stage (right), one asterisk (*) indicates a *P* value of 0.0110 (unpaired two-tailed Student's *t* test) for the comparison of the Δ*ssdA* mutant to the wild type and two asterisks (**) indicate a *P* value of 0.007 (unpaired two-tailed Student's *t* test) for the comparison of the OE:*ssdA* mutant to the wild type. (B and C) Radial growth assays were performed with each strain using GMM at 37°C for 72 h. (B) Images are representative of three independent experiments with similar results. (C) Measurement of the radial growth was performed at 72 h. Data represent means ± SE of results from three biological replicates. Three asterisks (***) indicate a *P* value of <0.0001 (unpaired two-tailed Student's *t* test compared to the wild-type CEA10 strain). (D) Fungal biomass was measured using 10^8^ spores in 100 ml liquid GMM at 37°C for 24 h. Data represent means ± SE of results from three biological replicates. One asterisk (*) indicates a *P* value of 0.0133, and two asterisks (**) indicate a *P* value of 0.0023 (unpaired two-tailed Student's *t* test). (E) Germination assays were utilized using 10^8^ spores in 10 ml liquid GMM at 37°C. A 500-μl volume of each culture was taken to count for the percentage of germlings at each time point. Data represent means ± SE of results from three biological replicates. Two asterisks (**) indicate a *P* value of *<*0.01 (unpaired two-tailed Student's *t* test comparing the *ssdA* null mutant to the wild type at 6 to 8 h). Three asterisks (***) indicate a *P* value of *<*0.0001 (unpaired two-tailed Student's *t* test comparing the overexpression strain to the wild type at 5 to 12 h).

To test this hypothesis, we generated an *ssdA* null (Δ*ssdA*) mutant and an overexpression (OE:*ssdA*) strain (confirmed by PCR and Southern blot analyses). The *ssdA* null mutant was complemented with an *ssdA*:*GFP* allele. We next measured the trehalose content of both conidia and mycelia in the respective strains (53). We observed increased trehalose content in Δ*ssdA* conidia and mycelia and, conversely, reduced trehalose levels in strain OE:*ssdA* (*P = *0.0004 [comparing the Δ*ssdA* mutant to the wild type] and *P < *0.0001 [comparing the OE:*ssdA* strain to the wild type]) (Fig. 1A). Reconstitution of strain Δ*ssdA* with *ssdA*:*GFP* restored wild-type trehalose levels. These data suggest that SsdA plays a role in regulating biosynthesis and/or levels of trehalose in A. fumigatus.

To determine if *ssdA* plays a role in germination of conidia and in polarized growth, we measured radial growth of fungal mycelia of the respective strains on solid medium (Fig. 1B and C) and liquid planktonic culture biomass (Fig. 1D) in 1% glucose minimal medium (GMM). We observed that both the Δ*ssdA* and OE:*ssdA* strains exhibited decreased mycelial radial growth on solid GMM after a 72-h incubation at 37°C compared to the wild-type and reconstituted strains (Δ*ssdA*+*ssdA*:*GFP*) (*P < *0.0001 [comparing strain Δ*ssdA* to the wild type] and *P = *0.0001 [comparing strain OE:*ssdA* to the wild type]) (Fig. 1B and C). In planktonic liquid cultures, the level of 24-h biomass from Δ*ssdA* and OE:*ssdA* cultures grown at 37°C was reduced compared to the wild-type and reconstituted strains (*P = *0.0133 [comparing strain Δ*ssdA* to the wild type] and *P = *0.0023 [comparing strain OE:*ssdA* to the wild type]) (Fig. 1D). Δ*ssdA* conidia germinated faster in the first 6 to 8 h (*P < *0.01 [comparing strain Δ*ssdA* to the wild type]), while OE:*ssdA* conidia germinated slower during the first 12 h and then caught up to the wild-type rate at 24 h (*P < *0.0001 [comparing strain OE:*ssdA* to the wild type]) (Fig. 1E). The reduced germination observed in OE:*ssdA* conidia is consistent with the reduction in trehalose levels in these fungal cells, as Al-Bader et al. observed that depletion of trehalose content in an A. fumigatus *tpsA*/*tpsB* double null mutant deficient in trehalose delayed germination of conidia (39). However, A. fumigatus trehalose mutants do not have *in vitro* growth defects when glucose is the primary carbon source, and we cannot attribute *ssdA* mutant growth defects to alterations in trehalose levels. Taken together, these results implicate SsdA in trehalose biosynthesis and support the idea of a global role for SsdA in fungal fitness when glucose is the sole carbon source.

### SsdA is important for cell wall integrity.

**FIG 2:**
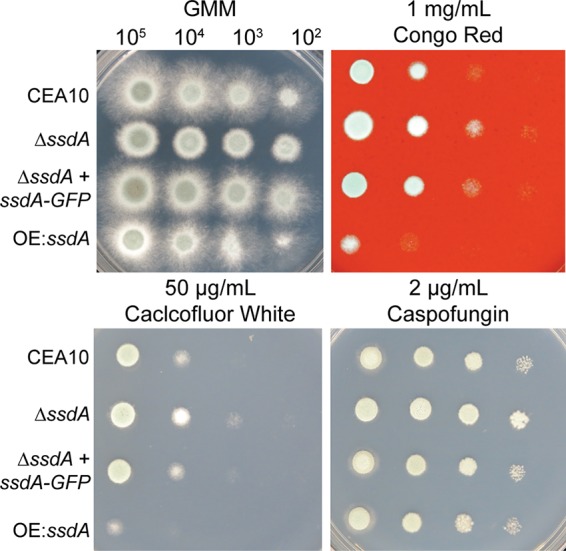
SsdA is important for cell wall integrity. Cell wall-perturbing agents, i.e., 1 mg/ml Congo red (upper right), 50 μg/ml calcofluor white (CFW) (lower left), and 2 μg/ml caspofungin (lower right), were utilized to study cell wall integrity in the respective strains. Cultures were incubated in the absence (upper left) or presence of the indicated agents at 37°C for 48 h. Data are representative of three independent experiments with similar results.

A. fumigatus trehalose mutants, including Δ*tslA* mutants, have altered cell wall integrity, and previous research identified a link between yeast Ssd1p and Neurospora crassa GUL-1 (SSD1 homolog) function and the cell wall (39, 40, 50, 54). We next utilized the cell wall-perturbing agents Congo red (1 mg/ml) and calcofluor white (CFW; 50 μg/ml) and the echinocandin caspofungin (CPG; 2 μg/ml) to test the hypothesis that A. fumigatus SsdA is important for cell wall integrity. Strain Δ*ssdA* exhibited increased resistance to cell wall-perturbing agents, while strain OE:*ssdA* exhibited increased susceptibility, particularly to Congo red and calcofluor white (Fig. 2).

**FIG 3:**
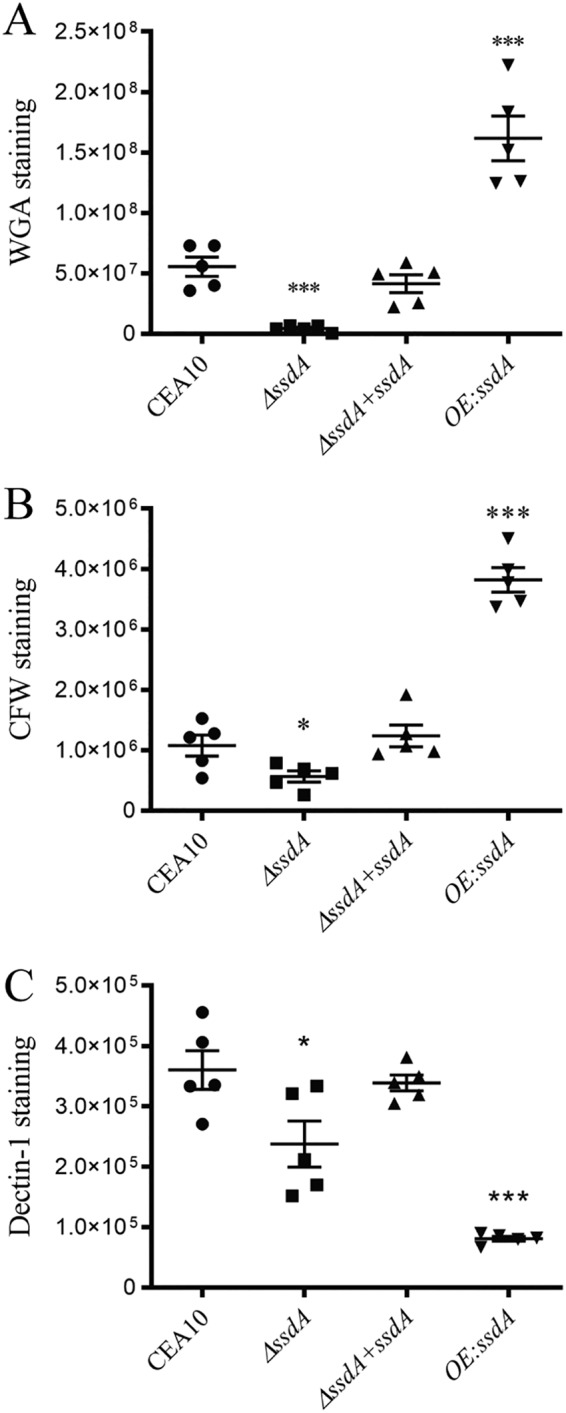
Alteration of *ssdA* expression affects exposure of cell wall PAMPs. Calcofluor white (CFW) staining (A), wheat germ agglutinin (WGA) treatment (B), and soluble dectin-1 (sDectin-1) staining (C) were utilized to observe chitin levels/exposure and β-glucan exposure on the cell wall of the respective strains. Each strain was cultured to the germling stage under normoxic conditions at 37°C. The corrected total cell fluorescence (CTCF) was calculated. For CFW staining, a single asterisk (*) indicates a *P* value of 0.0322 (unpaired two-tailed Student's *t* test comparing the Δ*ssdA* mutant to the wild type) and three asterisks (***) indicate a *P* value of *<*0.0001 (unpaired two-tailed Student's *t* test comparing the OE:*ssdA* to the wild type). For WGA staining, three asterisks (***) indicate a *P* value of 0.0002 (unpaired two-tailed Student's *t* test) comparing the Δ*ssdA* mutant to the wild type and a *P* value of 0.0008 (unpaired two-tailed Student's *t* test) comparing the OE:*ssdA* mutant to the wild type. For sDectin-1 staining, a single asterisk (*) indicates a *P* value of 0.0389 (unpaired two-tailed Student's *t* test comparing the Δ*ssdA* mutant to the wild type) and three asterisks (***) indicate a *P* value of *<*0.0001 (unpaired two-tailed Student's *t* test comparing the OE:*ssdA* mutant to the wild type). Data represent means ± SE for 15 images from three biological replicates. Bar, 3 μm. Data corresponding to the *y* axis represent arbitrary units (AU).

We hypothesized that the altered growth of and change in susceptibility to cell wall-perturbing agents observed in SsdA mutants come from altered cell wall composition and/or organization. To initially test this hypothesis, CFW and wheat germ agglutinin (WGA) were used to interrogate total and exposed chitin, respectively, while soluble human dectin-1-FC was used to examine β-1,3-glucan exposure. We observed a large decrease in the intensity of CFW and WGA staining on Δ*ssdA* germlings, whereas, germlings of OE:*ssdA* showed increased intensity with these chitin binding molecules (*P = *0.0322 [comparing strain Δ*ssdA* to the wild type] and *P < *0.0001 [comparing strain OE:*ssdA* to the wild type]) (Fig. 3A and B). For β-1,3-glucan, we observed a decrease in soluble dectin1-FC staining on both the Δ*ssdA* and the OE:*ssdA* germlings that was suggestive of a decrease in β-1,3-glucan exposure (*P = *0.0389 [comparing strain Δ*ssdA* to the wild type] and *P < *0.0001 [comparing strain OE:*ssdA* to the wild type]) (Fig. 3C). While additional quantitative cell wall composition analyses are needed, these data support the hypothesis that SsdA impacts A. fumigatus cell wall integrity.

**FIG 4:**
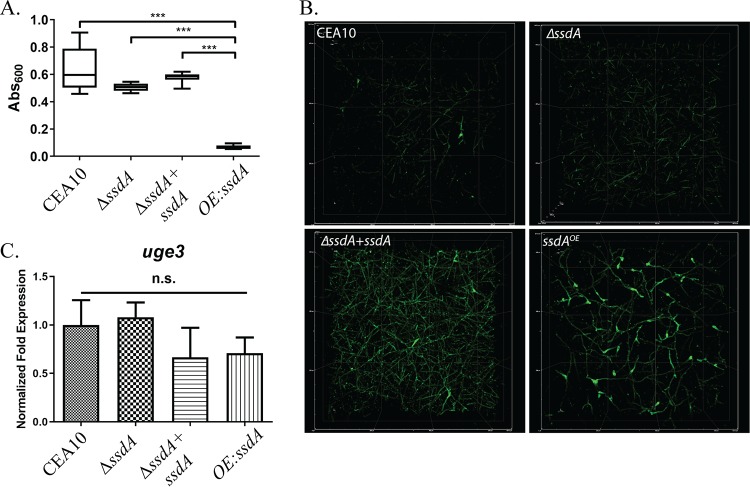
Expression of *ssdA* impacts adherence and biofilm formation. (A) Biofilms were grown at 37°C for 24 h in wells of a 96-well plate, and the crystal violet adherence assay was performed. Bars represent 6 replicates per strain, and the experiment was repeated 3 times with the same results. Three asterisks (***) indicate a *P* value of <0.0001 (one-way ANOVA with a Tukey posttest). (B) Micrographs of 24-h biofilms stained with FITC-conjugated soybean agglutinin. Images represent the top view of a Z-stack of the first 300 to 320 µm of the biofilm. Images are representative of 3 biological replicate cultures. (C) RNA was obtained from 24-h biofilm cultures, and qRT-PCR was performed for *uge3* mRNA levels. Data were normalized to *tef1* transcript levels. n.s., not significant by one-way ANOVA with a Tukey posttest.

Given the changes in the cell wall of the Δ*ssdA* and the OE:*ssdA* strains, we next tested their ability to adhere to an abiotic surface. Using the crystal violet adherence assay, we observed no differences in adherence among the wild-type, Δ*ssdA*, and reconstituted strains. However, a striking loss of adherence was observed in the OE:*ssdA* strain (Fig. 4A). To investigate this adherence difference further, spinning disk confocal microscopy, in combination with galactosaminogalactan binding fluorescein isothiocyanate (FITC)-labeled soy bean agglutinin (SBA), was utilized. Given the decreased adherence of the overexpression strain, we were surprised that increased expression of *ssdA* resulted in greater levels of SBA staining, revealing striking differences from the wild-type and Δ*ssdA* strains (Fig. 4B). As SBA binds to oligosaccharides with alpha- or beta-linked N-acetylgalactosamine and, to a lesser extent, galactose residues, we tested whether the mRNA levels of the UDP-glucose 4-epimerase involved in galactosaminogalactan biosynthesis were altered in the *ssdA* mutant strains (55). No significant differences in *uge3* mRNA levels were observed under the conditions examined, suggesting a role for SsdA in posttranscriptional regulation of the galactosaminogalactan polysaccharide (Fig. 4C). Taken together, these data suggest that *ssdA* expression levels impact fungal adherence.

**FIG 5:**
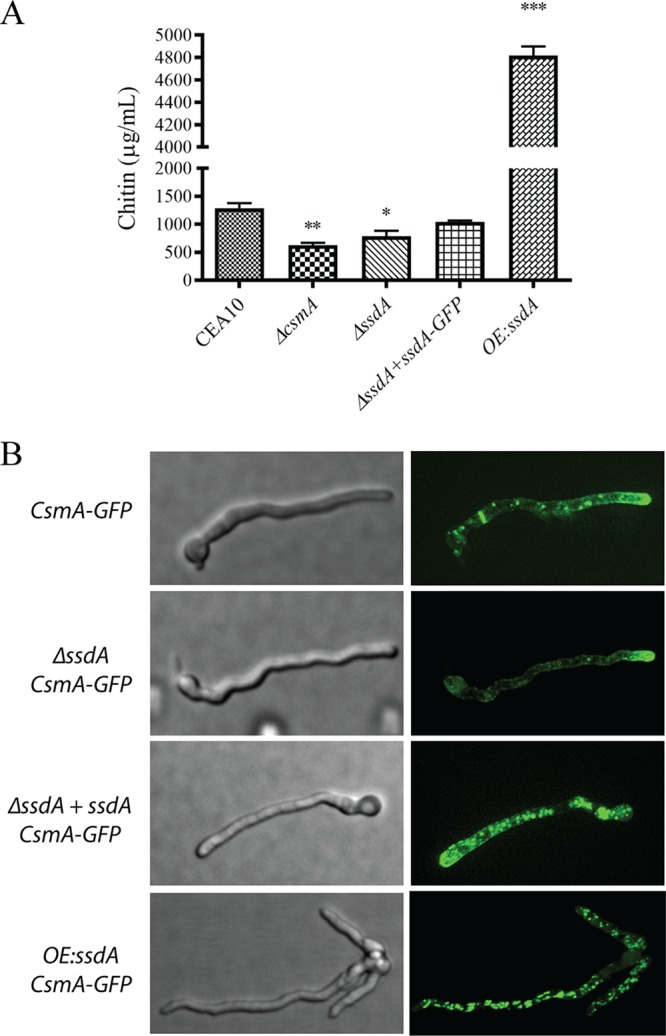
Chitin activity and CsmA localization. (A) A 10-μg volume of membrane proteins was used to perform a nonradioactive chitin synthase activity assay. Two asterisks (**) indicate a *P* value of 0.0029 (unpaired two-tailed Student's *t* test comparing the Δ*csmA* to the wild type); one asterisk (*) indicates a *P* value of 0.0208 (unpaired two-tailed Student's *t* test comparing the Δ*ssdA* mutant to the wild type); three asterisks (***) indicate a *P* value of <0.0001 (unpaired two-tailed Student's *t* test comparing the OE:*ssdA* mutant to the wild type). Data represent means ± SE of results from three biological replicates. (B) C-terminal GFP-tagged CsmA was generated in the wild-type and Δ*ssdA*, *ΔssdA+ssdA*, and OE:*ssdA* mutant backgrounds. Each strain was cultured at 37°C for 12 h, and live-cell imaging was performed under a Quorum Technologies WaveFX spinning disk confocal microscope (×1,000). The images were analyzed using Imaris 8.1.4 software. Data are representative of 15 images from three biological replicates. Bar, 3 μm.

Given the responses of the *ssdA* mutant strains to agents and reagents that inhibit or bind to chitin, a nonradioactive chitin synthase activity assay was next utilized to further define the impact of SsdA levels on the A. fumigatus cell wall (56, 57). Consistent with the cell wall immunohistochemistry results, chitin synthase activity in strain Δ*ssdA* was significantly reduced whereas, in contrast, chitin synthase activity in strain OE:*ssdA* was significantly increased (*P = *0.0029 [comparing strain Δ*csmA* to the wild type], *P = *0.0208 [comparing strain Δ*ssdA* to the wild type], and *P < *0.0001 [comparing strain OE:*ssdA* to the wild type]) (Fig. 5A). We previously observed that the activity and localization of the chitin synthase CsmA were perturbed by loss of the TslA trehalose regulatory protein (41). As TslA was found to also physically interact with SsdA, we hypothesized that SsdA levels may also impact CsmA subcellular localization.

To study CsmA subcellular localization under conditions in which SsdA levels are altered, we introduced a C-terminal green fluorescent protein (GFP)-tagged *csmA* allele into the respective *ssdA* mutant strains. Using spinning disk confocal microscopy, we observed that alteration (loss or increase) of *ssdA* mRNA levels led to an altered CsmA localization pattern compared to the wild-type and reconstituted strains (Fig. 5B). The CsmA:GFP puncta observed in strain Δ*ssdA* are mainly focused at the hyphal tip, with a few puncta also localized along the lateral hyphal walls but with no visible localization at the conidial septum. In contrast, in strain OE:*ssdA*, CsmA:GFP puncta were dispersed throughout the hyphae, with no visible puncta at the hyphal tip or conidial septum (Fig. 5B). Intriguingly, the latter result is similar to the diffuse subcellular localization of CsmA:GFP seen in the absence of TslA (41). Taken together, these results suggest that SsdA levels affect subcellular localization of the chitin synthase CsmA.

### SsdA levels are critical for Aspergillus fumigatus virulence.

**FIG 6:**
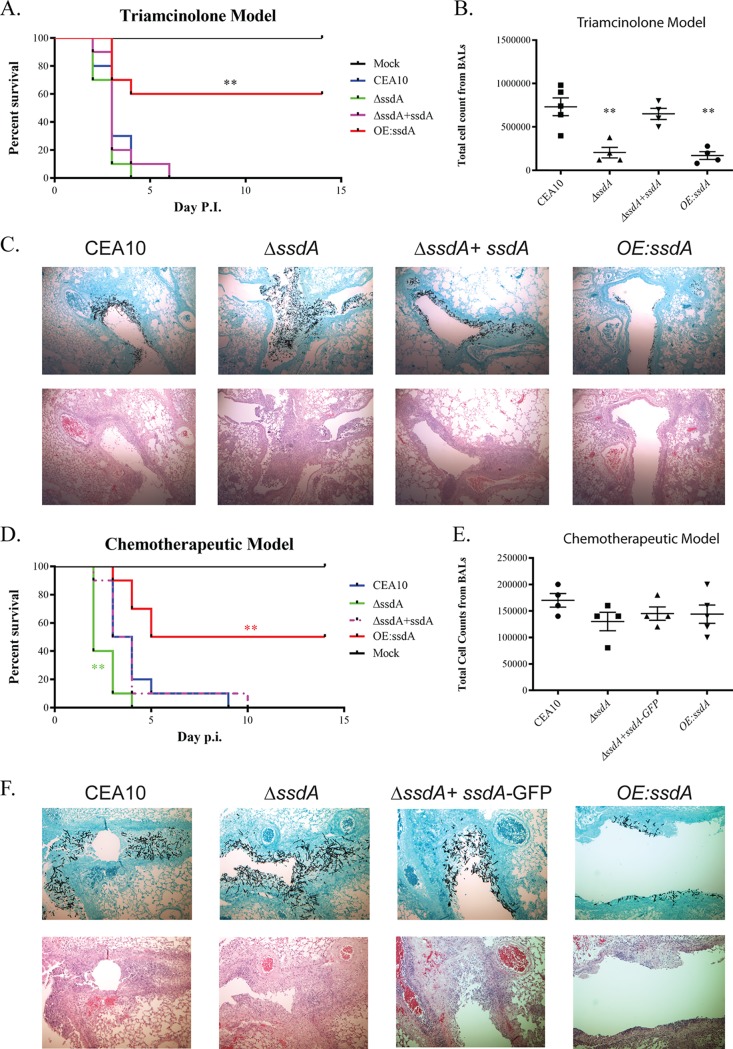
Overexpression of SsdA attenuates virulence in the triamcinolone murine model, while both loss of SsdA (Δ*ssdA*) and overexpression of SsdA (OE:*ssdA*) alter immune cell infiltrates. (A) A total of 2 × 10^6^ conidia of each strain were inoculated via the intranasal route in the triamcinolone IPA murine model. Ten CD1 mice were used in each group. Survival analysis was performed for 2 weeks. Two asterisks (**) indicate a *P* value of 0.0033 (log rank test comparing the OE:*ssdA* mutant to the wild type). P.I., postinfection. (B) Δ*ssdA* and OE:*ssdA*-infected BALs had decreased total levels of inflammatory cell infiltrations. For the total cell count data, a double asterisk (**) indicates a *P* value of 0.0159 comparing the wild type to the Δ*ssdA* mutant and OE:*ssdA* mutant, respectively, by a two-tailed Mann-Whitney *t* test. (C) OE:*ssdA* mutant-infected lungs show less fungal growth and less cell infiltration than those infected by the wild type. The fungal histology was performed on day 3 to observe fungal growth and inflammatory cell infiltration. GMS, Gomori-methenamine silver staining (top row); H&E, hematoxylin and eosin staining (bottom row). Magnification, ×50. (D) A total of 1 × 10^6^ conidia of each strain were inoculated intranasally for the chemotherapeutic murine model, and survival analyses were performed for 2 weeks using 10 CD1 mice per group. For the Δ*ssdA* mutant, two asterisks (**) indicate a *P* value of 0.005 (log rank test). For the OE:*ssdA* mutant, two asterisks (**) indicate a *P* value of 0.0049 (log rank test). (E) Δ*ssdA* and OE:*ssdA* mutant-infected BALs had total levels of inflammatory cell infiltrations similar to those seen with the wild-type BALs. (F) OE:*ssdA* mutant-infected lungs showed less fungal growth than those infected with the wild type. Histology was performed on day 3 to observe fungal growth and inflammatory cell infiltration. GMS, Gomori-methenamine silver staining (top row); H&E, hematoxylin and eosin staining (bottom row). Magnification, ×50.

Given the trehalose, cell wall, and biofilm phenotypes associated with alterations in SsdA levels, we hypothesized that SsdA plays an important role in A. fumigatus fungus-host interactions. To understand the importance of SsdA in the A. fumigatus-host interaction, we first utilized the triamcinolone (steroid) murine model of IPA (58). Strikingly, we observed that overexpression of *ssdA* significantly decreased A. fumigatus virulence compared to that seen with the wild type (*P* = 0.0033 [comparing strain OE:*ssdA* to the wild type]) (Fig. 6A). This reduction in virulence was associated with a large reduction in immune cell infiltrate in bronchoalveolar lavage fluid (BALF) samples (BALs) (*P = *0.0159 [comparing strain OE:*ssdA* to the wild type; Mann-Whitney *t* test]) (Fig. 6B). We also observed a significant reduction in fungal growth within the strain OE:*ssdA*-inoculated lungs compared to the results seen with the other strains (Fig. 6C). Taken together, the reductions in fungal burden and host immune cell infiltration likely explain the virulence defect of strain OE:*ssdA*.

In contrast, complete loss of SsdA did not alter median murine survival times as shown by comparisons between the wild type and Δ*ssdA* strains (median survival = 3 days). However, despite the *in vitro* growth defect of strain Δ*ssdA*, the fungal burden observed by histopathology revealed modest increases in strain Δ*ssdA* fungal burden at day 3 postinoculation compared to the wild type (Fig. 6C). It is possible that the observed *in vitro* increase in conidia germination of this strain contributed to the increase in fungal burden (Fig. 1E). Surprisingly, despite equivalent or increased levels of fungal burden compared to the wild type, a significant reduction in immune cell infiltrate in the bronchoalveolar lavage fluid (BALs) was apparent in animals inoculated with strain Δ*ssdA* (*P =* 0.0159 [comparing strain Δ*ssdA* to the wild type; Mann-Whitney *t* test]) (Fig. 6C). These results support the hypothesis that changes in *ssdA* levels impact the fitness of A. fumigatus *in vivo* and alter host immune responses.

Given the observed combination of a striking strain Δ*ssdA in vitro* growth defect and full virulence (as measured by murine mortality) in the steroid IPA model, we hypothesized that SsdA would be essential for virulence in a leukopenic IPA model with significant immune cell depletion (41). However, surprisingly, and similar to results observed with the triamcinolone model, strain Δ*ssdA* had persistent if not slightly increased virulence in the leukopenic model (*P *=* *0.005) (Fig. 6D). Also similar to results observed in the triamcinolone model, strain OE:*ssdA* had significant virulence attenuation compared to the wild type (*P = *0.0049) (Fig. 6D). The median durations of survival of the wild-type, Δ*ssdA*, and OE:*ssdA*-inoculated mice were 3.5, 2, and 9.5 days, respectively. Histopathology analysis applied to this leukopenic model revealed reduced fungal growth from lungs of OE:*ssdA*-inoculated mice whereas the Δ*ssdA*-inoculated mice, in contrast to the *in vitro* growth phenotype, had substantial invasive hyphal growth compared to the wild type (Fig. 6F). In contrast to the results observed with the triamcinolone model, the levels of inflammatory cell infiltrations were the same in comparisons between the *ssdA* mutants and the wild-type strain in this leukopenic model, possibly reflecting the significant chemical-mediated immune suppression (Fig. 6E). These results suggest that increased SsdA levels attenuate A. fumigatus virulence, likely through fungal fitness defects, while loss of SsdA alters the host immune response and modestly increases fungal virulence *in vivo*.

## DISCUSSION

The cell wall of Aspergillus fumigatus consists of polysaccharides, including chitin, β-glucan, and galactosaminogalactan and others that are critical for fungal fitness in diverse environments, including those associated with pathogenesis (29–38). Cell wall homeostasis and integrity are critical for the synthesis of each cell wall component in the face of stress and affect fungal pathogenesis on multiple levels (59). Another carbohydrate produced by fungi, trehalose, is also critical for fungal fitness during environmental stress, including pathogenesis (42, 60). Previous research in multiple fungi revealed an unexpected and ill-defined link between cell wall homeostasis and the biosynthesis of the disaccharide sugar trehalose (39–41). In A. fumigatus, a physical interaction between the TslA trehalose biosynthesis regulatory subunit and CsmA, a class V chitin synthase, suggested that trehalose biosynthesis proteins have direct roles in coordinating trehalose and fungal cell wall biosynthesis (41). Coordination between these 2 biological processes is logical given that the two biosynthetic pathways utilize common carbohydrate metabolic intermediates. Intriguingly, in our previous study, TslA was observed to physically interact with a protein (SsdA) that we define here as a homolog of the S. cerevisiae translational repressor protein Ssd1p. Alterations in the levels of SsdA in A. fumigatus impact both trehalose levels and cell wall integrity. Thus, these data further support the hypothesis that trehalose and cell wall biosynthesis are coordinated and implicate a new potential regulatory protein SsdA in these processes in A. fumigatus.

How physical interactions between TslA, SsdA, and CsmA in A. fumigatus mediate chitin and trehalose biosynthesis is unclear. In S. cerevisiae, Ssd1p is a unique RNA-binding protein associated with multiple biological processes (43, 44), including stress tolerance, membrane trafficking, cell cycle, posttranslational modifications, minichromosome stability, and cell wall integrity (45–47, 61). With regard to a regulatory role in cell wall biosynthesis in yeast, Hogan et al. showed that mRNA transcripts associated with Ssd1 encoded proteins related to cell wall biosynthesis, cell wall remodeling and regulation, the cell cycle, and protein trafficking (44). Loss of S. cerevisiae Ssd1p from both human and plant yeast isolates impacted cell wall composition by increasing both chitin content and mannan content while decreasing β-1,3-glucan content (50). Intriguingly, these results from yeast contrast with those observed here in A. fumigatus, where SsdA loss appears to decrease chitin content whereas overexpression of SsdA increased chitin content. However, additional cell wall composition biochemical assays are needed to define the impact of SsdA on cell wall composition.

Ssd1 homologs are also associated with cell wall integrity in the human-pathogenic yeast Cryptococcus neoformans, though a *ssd1* loss-of-function strain displayed only modest susceptibility to cell wall-perturbing agents in this pathogenic yeast (62). However, an Ssd1 homolog (*Ca*SSD1) in the human-pathogenic yeast Candida albicans is associated with cell wall integrity and virulence. Increased expression of *CaSSD1* is associated with antimicrobial peptide resistance, while *ssd1* deletion mutants exhibited decreased virulence in an invasive candidiasis murine model (51). Intriguingly, *Ca*Ssd1p physically interacts with *Ca*Cbk1p, an NDR (nuclear Dbf2-related) kinase, which is important for hyphal morphogenesis, the RAM (regulation of Ace2 and morphogenesis) pathway, polarized growth, cell proliferation, apoptosis, and cell wall biosynthesis (63, 64). *Ca*Ssd1p has nine *Ca*Cbk1p phosphorylation consensus motifs. *Ca*Cbk1p is essential for Ssd1p localization to polarized growth areas (63). Moreover, in the filamentous fungus Neurospora crassa, a *gul-1* (Ssd1 homolog) mutant was able to partially suppress the severe fitness defect of a *cot-1* (Cbk1 homolog) temperature-sensitive mutant and this was found to be associated with a reduction in transcript levels of cell wall homeostasis genes, including those encoding chitin synthases and the beta 1,3 glucan synthase *fks1* (54, 65).

The putative A. fumigatus Cbk1 homolog (AFUB_068890) is uncharacterized, but the corresponding homolog in A. nidulans, CotA, is a conditionally essential gene, and it is unclear if it plays a direct role in cell wall or trehalose biosynthesis (66–68). However, loss of A. nidulans *cotA* phenotypes can be suppressed by osmotic stabilization, perhaps suggesting an important role for this kinase in cell wall biosynthesis in *Aspergillus* spp. (68). Future experiments with *cotA* loss-of-function and/or gain-of-function mutants in A. fumigatus may reveal if this important kinase plays a role in chitin synthase regulation and whether such a role is mediated by TslA and/or SsdA. Additional domain-specific mutations in TslA/SsdA and/or genetic screens may also help reveal the mechanistic relationship(s) behind the TslA-SsdA protein-protein interaction and chitin biosynthesis.

Importantly for human fungal pathogenesis, our results indicate that A. fumigatus SsdA plays a role in virulence. Clinical and plant yeast isolates with null mutations in *Scssd1* were found to have increased virulence in a DBA/2 murine infection model (50). *Scssd1* null mutants induced more proinflammatory cytokine production, perhaps consistent with alterations in cell wall composition in Ssd1 mutants (50). In the fungal plant pathogens Colletotrichum lagenarium and Magnaporthe oryzae, *SSD1* is also important for pathogenesis (52). It was hypothesized that *SSD1* supported plant infection by evading induction of the plant immune response (52). Interestingly, the loss of SsdA in A. fumigatus resulted in a level of virulence similar to that seen with the wild-type strain as measured by murine mortality in both the triamcinolone and leukopenic murine IPA models despite the *in vitro* colony and planktonic growth defects associated with *ssdA* loss. In fact, in both murine models, loss of *ssdA* appeared to promote *in vivo* fungal growth but, intriguingly, reduced the host immune response. In contrast, overexpression of *ssdA* severely attenuated virulence and we observed significantly less fungal growth in the OE:*ssdA-*inoculated lungs, suggesting that loss of virulence in this strain may be due to poor *in vivo* fitness. The extreme adherence defect of strain OE:*ssdA* may contribute to this loss of *in vivo* fungal burden and virulence, but we cannot rule out the possibility of other mechanisms being impacted by increased SsdA levels. For example, the significant delay in germination of conidia observed in the OE:*ssdA* strain *in vitro* may also manifest *in vivo* and give the host immune system additional time to clear the fungus. Perhaps consistent with altered cell wall composition and pathogen-associated molecular pattern (PAMP) exposure, both Δ*ssdA*-inoculated and OE:*ssdA*-inoculated BALF samples had decreased inflammatory cell infiltration, particularly of neutrophils, and how these alterations in the host inflammatory response mediated infection outcomes in the presence and absence of SsdA requires further investigation.

In conclusion, we identified a critical role for A. fumigatus SsdA in cell wall homeostasis, trehalose production, and virulence. SsdA is involved in regulation of chitin biosynthesis and/or homeostasis in this fungus; however, the mechanisms of this regulation are unclear, and further investigation is needed to fully understand the roles and mechanisms of SsdA in A. fumigatus cell wall integrity and fungus-host interactions. While there is a clear conservation of a role for SsdA homologs in cell wall homeostasis in fungi, these data in A. fumigatus provide another example of altered wiring/functions of key master regulatory genes in pathogenic fungi compared to model organisms. It will be interesting and important to explore the regulation and function of these pathways across and within fungal species to identify broadly conserved mechanisms for potential therapeutic development.

## MATERIALS AND METHODS

### Fungal strains, media, and growth conditions.

Aspergillus fumigatus strain CEA17 strain (a uracil auxotroph strain lacking *pyrG* gene) was used to generate the *ssdA* null mutant (69). Glucose minimal medium (GMM) containing 1% glucose was used to grow the mutants along with a wild-type strain, CEA10 (CBS144.89), at 37°C with 5% CO_2_ if not stated otherwise (71). The conidia from each strain were collected in 0.01% Tween 80 after a 72-h incubation at 37°C with 5% CO_2_. Fresh conidia were used in all experiments.

### Strain construction and fungal transformation.

Gene replacements and reconstituted strains were generated as previously described (40, 58). PCR and Southern blottiong were used to confirm the mutant strains (40). Real-time reverse transcriptase PCR (RT-PCR) was used to confirm expression of the reintroduced gene and overexpressed strain (72). To generate the single null mutant, A. parasiticus *pyrG* from pJW24 was used as a selectable marker (73). To generate reconstituted strains of single null mutants, we utilized as a marker *ptrA*, which is a pyrithiamine resistance gene from A. oryzae (74). To generate GFP-tagged strains, we utilized a hygromycin resistance marker *hygB*, which is a hygromycin B phosphotransferase gene (75). In localization experiments, we generated C-terminal GFP-tagged CsmA in both the wild-type (CEA17) background and the Δ*ssdA* background by using *pyrG* and *ptrA* as selectable markers, respectively. After the constructs were generated, polyethylene glycol-mediated transformation of fungal protoplasts was performed as previously described (79). For the *ptrA* marker transformation, we added pyrithiamine hydrobromide (Sigma P0256) to GMM supplemented with 1.2 M sorbitol (SMM) at 0.1 mg/liter (74). For the *hygB* marker transformation, we recovered the strains containing the *hygB* marker by adding hygromycin B (Calbichem 400052) to 0.7% top SMM agar at 150 μg/ml the day after transformation (75).

### Germination assays and biomass assays.

A total of 10^8^ conidia of each strain were cultured in 10 ml liquid GMM (LGMM) at 37°C in three biological replicates. A 500-μl volume of each culture was taken to determine germling percentages at the indicated time points. For biomass assays, 10^8^ conidia in 100 ml LGMM were cultured for each strain for 24 h at 37°C in three biological replicates. The biomass was collected and lyophilized, and dry weight was recorded.

### Cell wall-perturbing agents and antifungal agents.

Several cell wall-perturbing agents were utilized for cell wall integrity tests as follows. Congo red (CR) (Sigma C6277), calcofluor white (CFW) (fluorescent brightener 28; Sigma F3543), and caspofungin (CPG) (Cancidas, Merck and Co., Inc.). CR, CFW, or CPG was added into GMM plates at a final concentration of 1 mg/ml, 50 μg/ml, or 1 μg/ml, respectively. Dropout assays were performed by plating serial conidial dilutions of 1 × 10^5^ to 1 × 10^2^ conidia in a 5-μl drop of each strain. The plates were cultured at 37°C with 5% CO_2_, and images were taken at 48 h. This experiment was performed in three biological replicates (40).

### Cell wall PAMP exposure.

Calcofluor white (CFW; 25 μg/ml), fluorescein-labeled wheat germ agglutinin (WGA; 5 μg/ml) (FL-1021; Vector Labs), and soluble dectin-1 staining were performed as previously described (80, 81). Briefly, each fungal strain was cultured on liquid glucose minimal media until it reached the germination stage. The hyphae were UV irradiated at 6,000 mJ/cm^2^. Z-stack micrographs were taken by the use of a Zeiss HAL 100 fluorescence microscope (Carl Zeiss Microscopy, LLC, Thornwood, NY, USA) equipped with a Zeiss Axiocam MRm camera. The intensity was analyzed using ImageJ, and the corrected total cell fluorescence (CTCF) was calculated (80, 82). Data represent means ± standard errors (SE) of data from 15 images from three biological replicates.

### Adherence assay and biofilm microscopy.

For the crystal violet adherence assay, 100 µl of 10^5^ spores per ml in GMM were inoculated into U-bottomed 96-well plates and grown for 24 h at 37°C. Plates were washed with H_2_O twice, stained with 0.1% (wt/vol) crystal violet in water for 10 min, washed twice more with H_2_O to remove excess stain, and destained with 100% ethanol for 10 min. Aliquots of the destained supernatants were transferred to a flat-bottomed 96-well plate, and absorbance at 600 nm (Abs_600_) was measured using a plate reader. Results were analyzed using one-way analysis of variance (ANOVA) with a Tukey posttest. For microscopy, 10^5^ spores per ml were grown in GMM for 24 h at 37°C on Mattek dishes (P35G-1.5-10-C). Biofilms were stained with 20 µg/ml FITC-SBA (FL-1011; Vector Labs) and fixed with 1% paraformaldehyde. Stained biofilms were imaged using a 20× multi-immersion objective on an Andor W1 spinning disk confocal microscope with a Nikon Eclipse Ti inverted microscope stand with Perfect Focus and equipped with two Andor Zyla cameras and an ASI MS-2000 stage. Z-stacks of the first 300 to 320 µm of the biofilm were taken for each sample. Microscopy was performed on three biological replicates per strain.

### RNA extraction and qRT-PCR.

RNA was extracted from 24-h biofilms grown at 37°C in GMM. Briefly, fungal tissue was flash frozen and bead beaten with 2.3-mm-diameter zirconia/silica beads in 200 µl of Bioline TriSure (BIO-38032). Homogenized mycelia were brought to a final volume of 1 ml, and RNA was processed according to manufacturer’s instructions. For reverse transcriptase quantitative PCR (qRT-PCR), 5 μg of RNA was subjected to DNase treatment with Ambion Turbo DNase (Life Technologies) according to the manufacturer’s instructions. For qRT-PCR, DNase-treated RNA was processed as previously described (83). mRNA levels were normalized to *tef1* levels for all qRT-PCR analyses. Statistical analysis was performed with one-way ANOVA with Tukey posttest. Error bars indicate standard deviations (SD) of the means.

### Chitin synthase activity assay.

A total of 10^8^ conidia of each fungal strain were grown at 37°C for 24 h in 10 ml of liquid GMM at 250 rpm. The mycelia were collected to prepare membrane fractions by centrifugation at 100,000 × g for 40 min at 4°C as described above. After that, the nonradioactive chitin synthase activity assay was performed in a 96-well plate as previously described (56, 57).

### Trehalose measurement.

Trehalose content in conidia and mycelia was determined as previously described (40). Briefly, A. fumigatus strains were grown on GMM plates at 37°C for 3 days. A total of 2 × 10^8^ conidia were used for the conidial stage of the trehalose assay, and 1 × 10^8^ conidia were cultured in 10 ml LGMM overnight for the mycelial stage as described by d’Enfert and Fontaine (53). Cell-free extracts were then tested for trehalose levels according to the protocols of a glucose assay kit (Sigma AGO20). Results from biological triplicate experiments were averaged, standard deviation calculated, and statistical significance determined (*P <* 0.05) with a two-tailed Student's *t* test.

### Murine models of invasive pulmonary aspergillosis.

CD1 female mice, 6 to 8 weeks old, were used in the triamcinolone (steroid) or the chemotherapeutic murine model experiments as previously described (40, 41, 58). Mice were obtained from Charles River Laboratories (Raleigh, NC). For survival studies and histopathology, 10 mice per A. fumigatus strain (including strains CEA10, Δ*ssdA*, *ΔssdA+ssdA-GFP*, and OE:*ssdA*) were inoculated intranasally with 2 × 10^6^ conidia in 40 μl of phosphate-buffered saline (PBS) for the triamcinolone model and 1 × 10^6^ conidia for the chemotherapeutic model and were monitored three times a day. Mice were observed for 14 days after the A. fumigatus challenge. Any animals showing distress were immediately humanely sacrificed and represented in the study data as deaths within 24 h. No mock-inoculated animals perished. Statistical comparisons of the associated Kaplan-Meier curves were conducted with log rank tests (84). Lungs from all mice sacrificed at different time points during the experiment were removed for differential cell count and histopathology.

### Histopathology.

Three mice from each group (including the CEA10, Δ*ssdA*, *ΔssdA+ssdA-GFP*, and OE:*ssdA* groups) were humanely euthanized at day 3 postinoculation. Lungs were harvested from each group and fixed in 10% formalin before embedding in paraffin was performed. Sections (5 μm thick) were taken and stained with either H&E (hematoxylin and eosin stain) or GMS (Gomori-methenamine silver stain) as previously described (85). The microscopic examination was performed using a Zeiss Axioplan II microscope and an engaged imaging system. Images were captured at ×50 magnification as indicated in each image.

### Collection and analysis of bronchoalveolar lavage fluid (BALF).

At the indicated time after A. fumigatus instillation, mice were euthanized using CO_2_. Bronchoalveolar lavage fluid (BALF) was collected by washing the lungs with 2 ml of PBS containing 0.05 M EDTA. BALF was then centrifuged and the supernatant collected and stored at −20°C until analysis. BAL fluid cells were resuspended in 200 µl of PBS and counted on a hemocytometer to determine total cell counts. Cells were then spun onto glass slides using a Thermo Scientific Cytospin4 cytocentrifuge and subsequently stained with a Diff-Quik staining kit (Electron Microscopy Sciences) for differential cell counting (80).

### Ethics statement.

This study was carried out in strict accordance with the recommendations in the Guide for the Care and Use of Laboratory Animals of the National Institutes of Health. The animal experimental protocol was approved by the Institutional Animal Care and Use Committee (IACUC) at Dartmouth College (protocol number cram.ra.1).
